# Crystallization of Feline Coronavirus M^pro^ With GC376 Reveals Mechanism of Inhibition

**DOI:** 10.3389/fchem.2022.852210

**Published:** 2022-02-24

**Authors:** Jimmy Lu, Sizhu Amelia Chen, Muhammad Bashir Khan, Raelynn Brassard, Elena Arutyunova, Tess Lamer, Wayne Vuong, Conrad Fischer, Howard S. Young, John C. Vederas, M. Joanne Lemieux

**Affiliations:** ^1^ Department of Biochemistry, University of Alberta, Edmonton, AB, Canada; ^2^ Li Ka Shing Institute of Virology, University of Alberta, Edmonton, AB, Canada; ^3^ Department of Chemistry, University of Alberta, Edmonton, AB, Canada

**Keywords:** 3CLpro, coronavirus, feline infectious peritonitis (FIP), FCoV, protease, GC376, antiviral, COVID-19

## Abstract

Coronaviruses infect a variety of hosts in the animal kingdom, and while each virus is taxonomically different, they all infect their host *via* the same mechanism. The coronavirus main protease (M^pro^, also called 3CL^pro^), is an attractive target for drug development due to its essential role in mediating viral replication and transcription. An M^pro^ inhibitor, GC376, has been shown to treat feline infectious peritonitis (FIP), a fatal infection in cats caused by internal mutations in the feline enteric coronavirus (FECV). Recently, our lab demonstrated that the feline drug, GC373, and prodrug, GC376, are potent inhibitors of SARS-CoV-2 M^pro^ and solved the structures in complex with the drugs; however, no crystal structures of the FIP virus (FIPV) M^pro^ with the feline drugs have been published so far. Here, we present crystal structures of FIPV M^pro^-GC373/GC376 complexes, revealing the inhibitors covalently bound to Cys144 in the active site, similar to SARS-CoV-2 M^pro^. Additionally, GC376 has a higher affinity for FIPV M^pro^ with lower nanomolar K_i_ values compared to SARS-CoV and SARS-CoV-2 M^pro^. We also show that improved derivatives of GC376 have higher potency for FIPV M^pro^. Since GC373 and GC376 represent strong starting points for structure-guided drug design, determining the crystal structures of FIPV M^pro^ with these inhibitors are important steps in drug optimization and structure-based broad-spectrum antiviral drug discovery.

## Introduction

Coronaviruses are single-stranded, positive-sense RNA viruses that affect mammals and birds, causing a variety of diseases (Anand et al., 2003). Containing one of the largest genomes among RNA viruses (∼27–31 kb), the family Coronaviridae makes up four genera: Alpha-, Beta-, Gamma-, and Deltacoronavirus (Báez-Santos et al., 2015). Coronaviruses take over the host's transcriptional machinery by encoding two overlapping polyproteins, pp1a and pp1ab, which are cleaved by coronavirus-encoded proteases—papain-like protease (PL^pro^) and main protease (M^pro^, also called 3CL^pro^) (Thiel et al., 2003)—forming 16 nonstructural proteins (nsps) that are essential for viral replication (de Wit et al., 2016). M^pro^, a cysteine protease, cleaves the polyproteins at 11 conserved sites containing the Leu-Gln↓(Ser, Ala, Gly) sequence, releasing the nsps required for the viral replicase complex (Hegyi and Ziebuhr, 2002). Since M^pro^ cleavage is required for subsequent viral replication and transcription, M^pro^ is an attractive target for drug development against coronaviruses (Yin et al., 2007).

Due to their rapid transmission and lethality, coronaviruses pose a major threat to public health (Chen et al., 2020). This was seen in previous global coronavirus outbreaks such as the severe acute respiratory syndrome coronavirus (SARS-CoV) outbreak in 2002/3, the Middle East respiratory syndrome coronavirus (MERS-CoV) outbreak of 2012, and more recently, the COVID-19 pandemic caused by SARS-CoV-2 in 2019 and onward (de Wit et al., 2016; Hu et al., 2021). Aside from humans, coronaviruses infect other mammals including felines, ferrets, mink, and pigs (Perera et al., 2018; Stout et al., 2020; Ye et al., 2020). Feline enteric coronavirus (FECV), an Alphacoronavirus, is commonly found among domestic cats; however, infected cats are usually asymptomatic or experience mild enteritis (Felten and Hartmann, 2019). Feline infectious peritonitis (FIP) derives from internal mutations in FECV shifting tropism from enterocytes to macrophages resulting in a 100% fatality rate in cats, thus FIP virus (FIPV) is vertically transmitted (Dye & Siddell, 2005; Felten and Hartmann, 2019). It is worth noting that mutations in FECV M^pro^ have not been associated with increased virulence in FIPV (Pedersen, 2014). M^pro^ inhibitors which block viral replication have, therefore, been extensively studied against different coronaviruses as a means to develop broad-spectrum antivirals (Hegyi and Ziebuhr, 2002; Kim et al., 2012; Bai et al., 2021).

Various peptidomimetic inhibitors have been developed against viral M^pro^ (Yin et al., 2007; St. John et al., 2015; Wang et al., 2016). In 2011, a peptide-based inhibitor of M^pro^ was reported as a promising antiviral drug to combat norovirus infection (Tiew et al., 2011). That inhibitor has since been modified and the new derivative, GC376, was shown to inhibit FIPV M^pro^ with sub-micromolar IC_50_ values (Kim et al., 2012). GC376, a dipeptidyl aldehyde bisulfite adduct, is a prodrug that converts into the active-form aldehyde, GC373, upon administration, effectively binding the active site of M^pro^ and stopping viral replication (Kim et al., 2013; Kim et al., 2015). Other studies have demonstrated that GC376 was successful in reversing the progression of experimentally induced FIP as well as naturally occurring FIP in cats, demonstrating that peptide-based inhibitors are effective against coronavirus infections *in vivo* (Kim et al., 2016; Pedersen et al., 2018).

With the success GC376 has had in treating FIP in cats, it was then postulated to be an effective inhibitor to treat SARS-CoV-2 infections (Vuong et al., 2020). We have previously reported that the prodrug GC376 and drug GC373 are potent inhibitors of SARS-CoV and SARS-CoV-2 M^pro^ with K_i_ values in the nanomolar range (Arutyunova et al., 2021). The crystal structures of SARS-CoV and SARS-CoV-2 M^pro^ in complex with the feline drugs revealed the inhibitor forming a covalent bond with Cys145 as a hemithioacetal in the active site (Vuong et al., 2020). The varied effectiveness of GC376 against M^pro^ of different coronaviruses suggests structural differences in drug binding (Kim et al., 2012; Arutyunova et al., 2021). Despite the research invested in the feline drugs with regard to FIP, no crystal structure of FIPV M^pro^ with GC376 or GC373 has been solved to date.

In this study, we solved the crystal structure of FIPV M^pro^ (FIPV WSU-79/1146) in complex with the drug, GC373, and prodrug, GC376, to reveal the architecture of the active site with bound inhibitors. Furthermore, we examined the improved derivatives of GC376 and demonstrated their higher potency toward FIPV M^pro^. As GC376 and GC373 were successfully used to treat FIP in cats, they are considered strong starting points in drug design to treat COVID-19 in humans. Here, we compare the structural similarities and differences between SARS-CoV-2 M^pro^ and FIPV M^pro^ with the feline drugs for antiviral drug optimization against SARS-CoV-2 and the development of future broad-spectrum antivirals.

## Materials and Methods

### Inhibitor and Fluorescence Resonance Energy Transfer Substrate Synthesis

Inhibitors GC373, GC376, and their derivatives, as well as the FRET assay peptide substrate, Abz-SVTLQSG-Y(NO2)-R, were synthesized according to methods previously described (Vuong et al., 2020; Vuong et al., 2021).

### Cloning, Expression, and Purification of Feline Infectious Peritonitis Virus M^pro^

The FIPV WSU-79/1146 M^pro^ gene was synthesized (Bio Basic, Canada) and cloned into pET SUMO expression vector (Invitrogen, United States), generating a fusion protein with a His-tagged SUMO domain at the N-terminus. The construct was transformed into BL21 (DE3) *Escherichia coli*, where protein expression was induced with 0.5 mM isopropyl β-D-1-thiogalactopyranoside (IPTG) once OD_600_ reached 0.5–0.6 and then grown for an additional 5 h at 32°C. The cells were harvested by centrifugation (5,000 ×*g* for 20 min at 4°C), suspended in lysis buffer (20 mM Tris-HCl, 150 mM NaCl, 5 mM imidazole, pH 7.8), and lysed using the Emulsiflex C3 High Pressure Homogenizer. Cellular debris was removed by centrifugation at 20,000 ×*g* for 45 min at 4°C. The isolated supernatant was applied onto a Ni-NTA column (Qiagen, Canada), the resin was washed with 10 column volumes of lysis buffer containing 20 mM imidazole, and the protein was eluted with a step gradient of 100–1000 mM imidazole in lysis buffer. The eluted fractions were analyzed by sodium dodecyl sulfate–polyacrylamide gel electrophoresis, pooled based on purity and dialyzed against 20 mM Tris-HCl, 150 mM NaCl, 1 mM TCEP, pH 7.8, for 2 h at 4°C. The SUMO tag was cleaved off using His-tagged SUMO protease (McLab, United States) and both the N-terminal SUMO tag and SUMO protease were removed by passing the protein sample through a Ni-NTA column. The flow-through containing FIPV M^pro^ was further purified using size exclusion chromatography (Superdex increase 10/300 GL, GE Healthcare), with buffer containing 20 mM Tris-HCl, 150 mM NaCl, 1 mM TCEP, pH 7.8. The fractions containing FIPV M^pro^ were pooled and concentrated using an Amicon Ultra-15 filter with a MWCO of 10 kDa.

### Crystallization of Feline Infectious Peritonitis Virus M^pro^ With GC373 and GC376

Purified FIPV M^pro^ was dialyzed against 5 mM Tris-HCl, 5 mM NaCl, 1 mM TCEP, pH 7.8 buffer at 4°C overnight and concentrated to 10 mg/ml using an Amicon Ultra-15 filter with a MWCO of 10 kDa. FIPV M^pro^ was incubated with GC373 or GC376 (5× molar excess) at 4°C for 2 h prior to crystallization. The protein was subjected to the PACT and JCSG crystallization screens (Molecular Dimensions, United States). Crystals were observed with sitting drop trays at room temperature. The crystals of FIPV M^pro^ with GC376 were obtained using a protein:buffer ratio of 1:1 with 2.0 M ammonium sulfate, 0.1 M Bis-Tris, pH 5.5. The crystals of FIPV M^pro^ with GC373 were obtained using a 2:1, protein:buffer ratio with 0.2 M calcium chloride dihydrate, 0.1 M MES, 20% (w/v) PEG 6000, pH 6.0. The crystals were frozen in liquid nitrogen using 19% glycerol as a cryoprotectant.

### Diffraction Data Collection, Model Building, and Structural Refinement

The diffraction data were collected at Canadian Light Source using beamline CMCF-BM (08B1) and PILATUS3 S 6M detector, Saskatchewan, Canada. Several data sets were collected from different crystals and were processed using SCALA and XDS. The diffraction data set of the GC373 was processed to 2.05 Å, in a monoclinic C2 space group, while the GC376 were processed to 1.93 Å, in an orthorhombic P2_1_2_1_2_1_ space group. The structures were determined by molecular replacement using the crystal structure of the apo-FIPV Mpro (PDB entry: 5EU8) as the search model. GC376 and GC373 were manually fit in the density using Coot. The structures were then refined by using the Phenix software. Data statistics, processing, and model refinement are given in Supplementary Table S1.

### Enzyme Kinetics of Feline Infectious Peritonitis Virus M^pro^

A fluorescence resonance energy transfer (FRET)–based cleavage assay was performed using a synthetic peptide fluorescent substrate containing the cleavage site of FIPV M^pro^ [Abz-SVTLQ↓SG-Tyr (NO2)-R] as described previously (Vuong et al., 2020; Vuong et al., 2021). For K_i_ determination, 50 nM FIPV M^pro^ was preincubated with GC376 in the concentration range of 0.01–0.4 µM for 10 min at 37°C. The enzymatic reactions using 1–500 μM of FRET substrate in activity buffer (25 mM Bis-Tris, 1 mM DTT, pH 7.0) were started with the addition of protease. For IC_50_ determination, 100 nM of FIPV M^pro^ was incubated with an inhibitor concentration range of 0.25 nM–100 µM in activity buffer. The reaction was started with 40 µM of FRET substrate. The fluorescence signal of the FRET peptide cleavage product was monitored at an emission wavelength of 420 nm with excitation at 320 nm, using a Cytation 5 Imaging Multi-Mode Reader (BioTek) for 7 min at 37°C. The kinetic data were analyzed using computer-fit calculation (Prism 9.0, GraphPad Software). The slopes of the Lineweaver–Burk plots were plotted versus the concentration of GC376, and the K_i_ was determined from the x-axis intercept as −K_i_. The experiments were performed in triplicate.

## Results

### The Overview of Feline Infectious Peritonitis Virus M^pro^-GC373/376 Complex Structure

**FIGURE 1:**
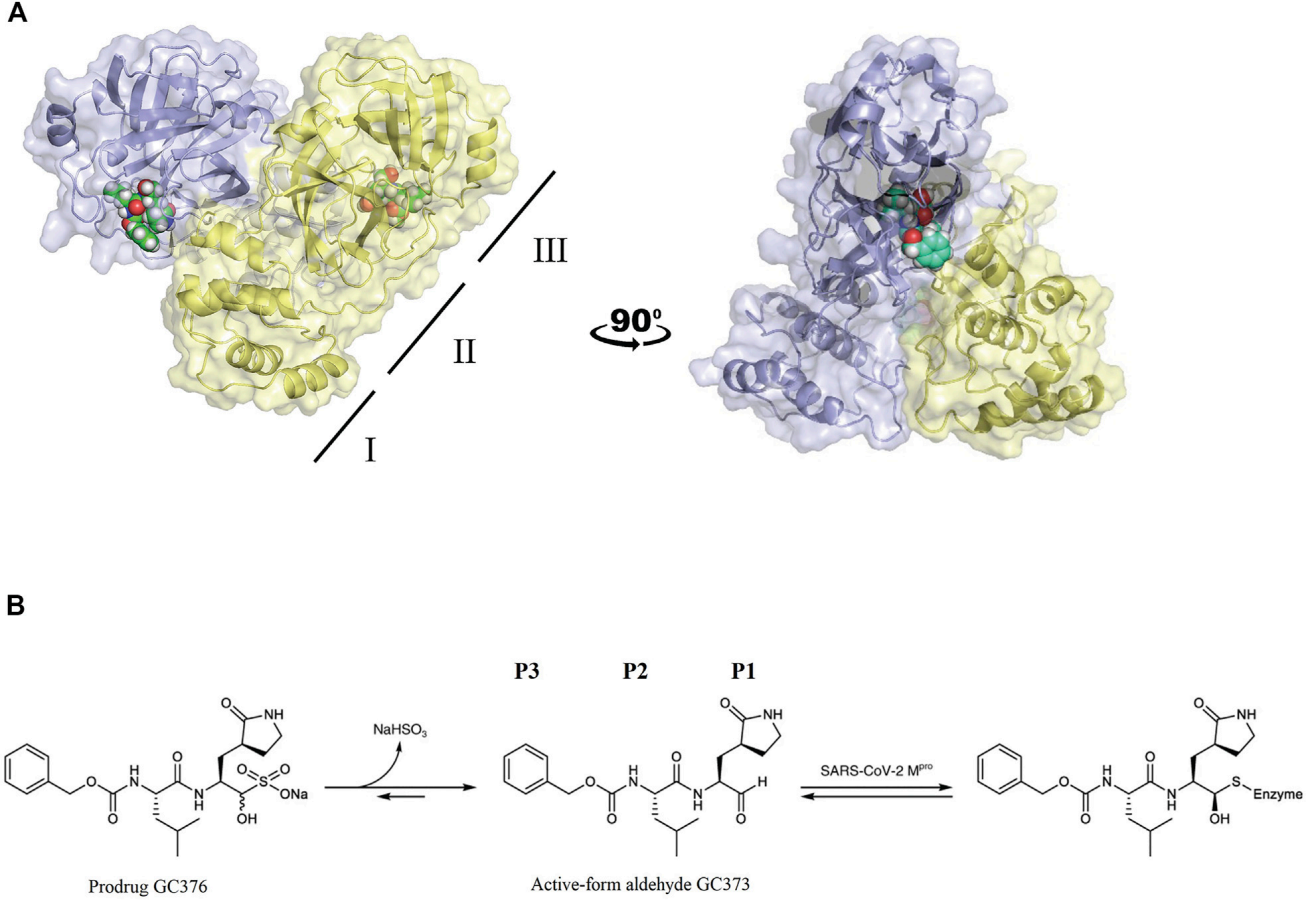
**(A)** FIPV M^pro^ exists as a dimer when bound with the feline drug GC373 (PDB: 7SNA). Domains I, II, and III are labeled on the left. Active sites of both protomers are occupied by GC373. **(B)** The prodrug, GC376, is a dipeptidyl aldehyde bisulfite adduct that readily converts into GC373 under aqueous conditions. GC373 covalently binds to the catalytic Cys144 of FIPV M^pro^.

Crystal structures of FIPV M^pro^ in complex with the drug GC373 (PDB: 7SNA) and prodrug GC376 (PDB: 7SMV) were solved to 2.05 Å and 1.93 Å, respectively (Supplementary Table S1). GC376 being the dipeptidyl aldehyde bisulfite adduct form of the drug converts into the active-form aldehyde GC373, thus making both structures identical. In both structures, FIPV M^pro^ crystallized as a dimer, with each protomer being comprised of three domains (Figure 1A), similar to other viral M^pro^. Domains I and II have a six-stranded antiparallel β-barrel structure, and domain III is a globular cluster of five antiparallel α-helices, connected to domain II by a long loop. The active site of FIPV M^pro^ contains a Cys144–His41 catalytic dyad located in a cleft between domain I and domain II. Domain III regulates the dimerization of the M^pro^ which is required for its catalytic activity. The N-terminal residues (N-finger) of protomer A fits between domains II and III of the protomer A and interacts with residues in domain II of protomer B helping shape the S1 substrate-binding subsite in the active site.

### GC373 Is Stabilized by H-Bond Network in the Active Site of Feline Infectious Peritonitis Virus M^pro^

**FIGURE 2:**
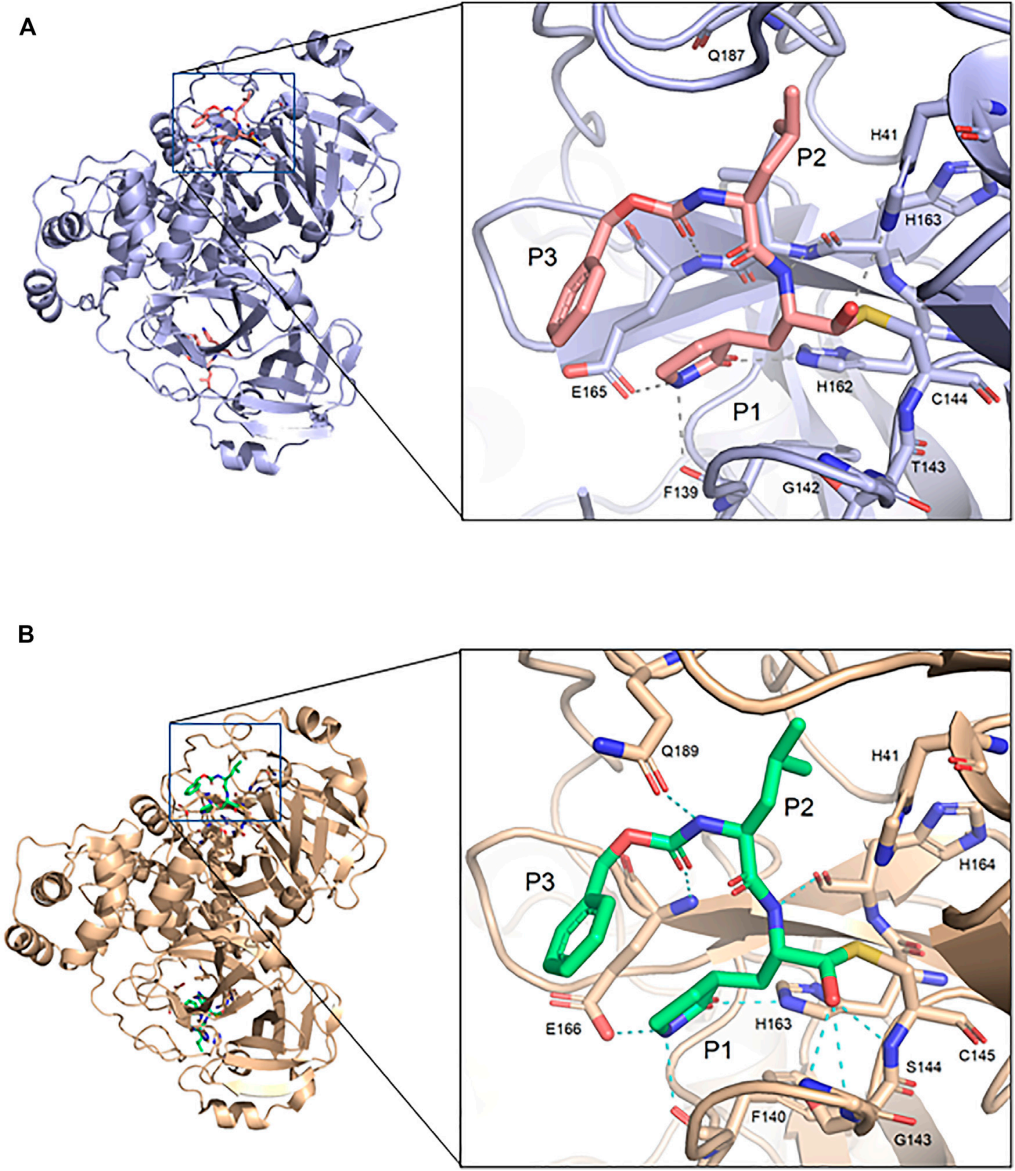
Comparison of FIPV M^pro^ and SARS-CoV-2 M^pro^ bound to GC373. **(A)** Crystal structure of FIPV M^pro^ with GC373 in lavender (PDB: 7SNA) and **(B)** SARS-CoV-2 M^pro^ with GC373 in tan (PDB: 6WTK).

The GC373 inhibitor covalently binds FIPV M^pro^ and is stabilized by hydrogen bonding and hydrophobic interactions in a similar manner to SARS-CoV and SARS-CoV-2 M^pro^. In both structures, a covalent bond between Cys144 and the aldehyde of the feline drug reveals that the bisulfite leaving group indeed was removed upon binding (Figure 1B). Weak H-bonding was observed between the oxyanion of the inhibitor and His41, the general base in the catalytic dyad, which is distinct from SARS-CoV-2 (Figure 2A) (Vuong et al., 2020). For the P1 position of the inhibitor, the Nγ of the lactam ring sits in the S1 pocket and forms a H-bond with the carbonyl oxygen of Phe139 (Supplementary Figure S1A), a conserved feature in other M^pro^ structures with GC373 (Arutyunova et al., 2021). The S2 pocket that supports hydrophobic interactions of a Leu moiety is formed with His41, Ile51, and Leu164 (Supplementary Figure S2A). This differs from the stabilization network found in SARS-CoV-2 M^pro^ for the same feline drug. Meanwhile, the P3 benzyl moiety interacts with the P1 lactam ring by pi stacking, similar to that observed in the SARS-CoV-2 M^pro^ structure (Vuong et al., 2020).

### Feline Infectious Peritonitis Virus M^pro^ and SARS-CoV-2 M^pro^ Have Similar Overall Structure

**FIGURE 3:**
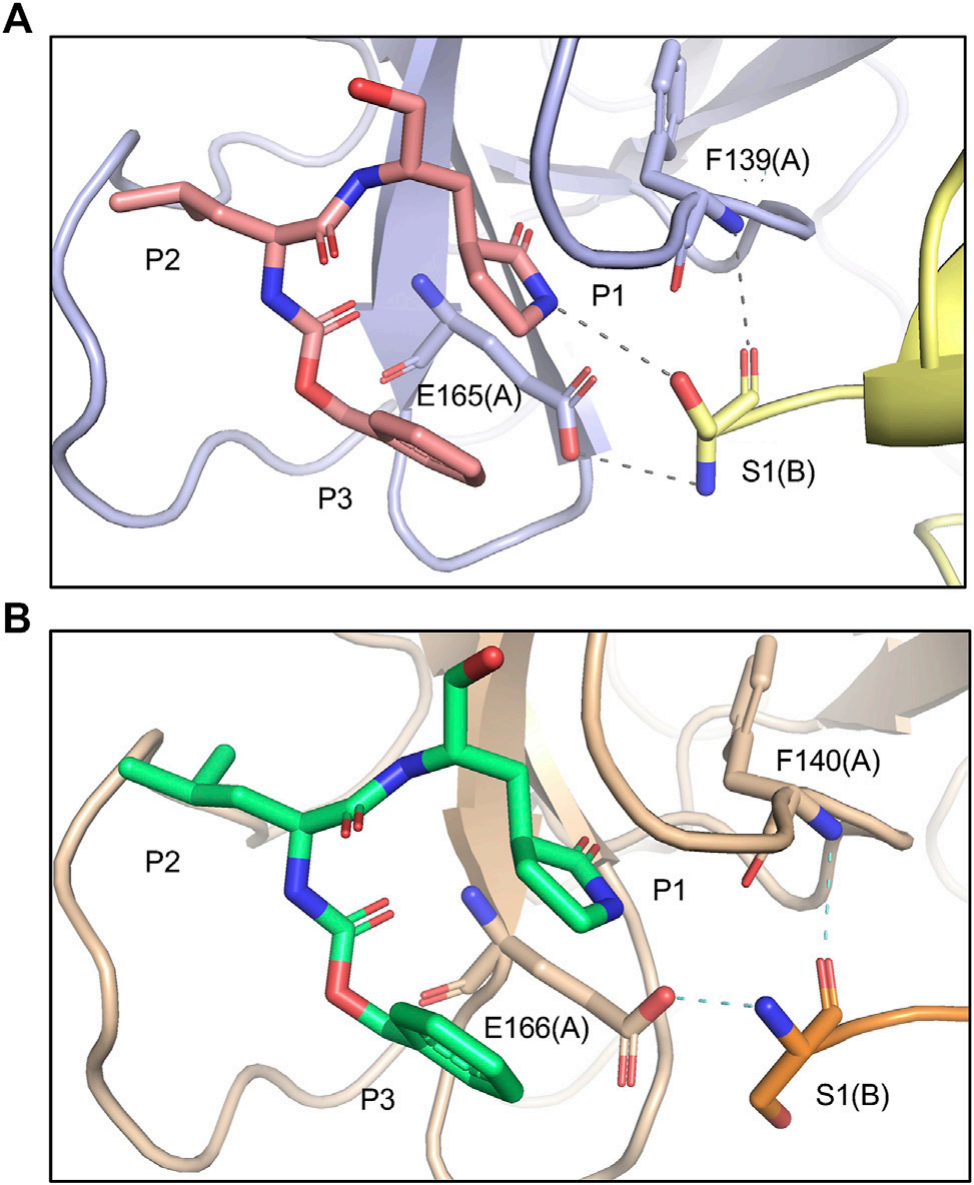
Comparing M^pro^ N-terminal fingers and their respective interaction with GC373. **(A)** In FIPV M^pro^ (PDB: 7SNA), Ser1 of the N-terminal finger from protomer B forms a weak H-bond (3.8 Å) with the cyclic glutamine analog nitrogen of GC373, as well as other H-bonds with E165 and F139 in the active site of protomer A. **(B)** In SARS-CoV-2 M^pro^ (PDB: 6WTK), Ser1 of the N-terminal finger from protomer B only forms H-bonds with E166 and F140 in the active site of protomer A but does not form H-bonds with GC373.

FIPV and SARS-CoV-2 M^pro^ share 60% sequence similarity, however, the active-site region exhibits even greater conservation (Supplementary Figure S3). Comparing the overall structures, both bound to GC373, the RMSD was calculated to be 1.16 Å. While the active site cavity of both FIPV and SARS-CoV-2 M^pro^ are composed of identical residues, the structures of M^pro^ in complex with GC373 or GC376, reveal some differences in inhibitor binding. In FIPV M^pro^, GC373 is stabilized in the active site by H-bonding with His41 (Figure 2A, Supplementary Figure S1B). By contrast, SARS-CoV (not shown) and SARS-CoV-2 M^pro^ form a stable acyl-intermediate with the drug through a H-bonding network with the backbones of Cys145, Ser144, and Gly143 residues (Figure 2B, Supplementary Figure S6). Furthermore, the S2 pocket that supports hydrophobic interactions of the drug's Leu moiety is formed with His41, Ile51, and Leu164 in FIPV M^pro^, but with His41, Met49, and Met165 in SARS-CoV-2 M^pro^ (Supplementary Figure S2B). In SARS-CoV-2 M^pro^, Gln189 plays an integral role in stabilizing the dipeptide backbone of the inhibitor (Bai et al., 2021), however in FIPV M^pro^, we observe an unstructured loop fit between the S3 and S4 pocket to form hydrophobic interactions, thus further supporting binding of the inhibitor (Supplementary Figures S4, S5). In FIPV M^pro^, Ser1 of the N-terminal finger from protomer B forms a weak H-bond (3.8 Å) with the cyclic glutamine analog nitrogen of GC373 in the active site of protomer A, however, this is not seen in SARS-CoV-2 M^pro^ (Figure 3A). Furthermore, the side chain hydroxyl group and backbone amide of Ser1 in protomer B form H-bonds with Glu165 and Phe139 in the active site of protomer A. This is comparable to SARS-CoV and SARS-CoV-2 M^pro^ structures where Ser1 of the N-terminal finger (protomer B) forms H-bonds with Glu166 and Phe140 (protomer A) to shape the P1 position (Figure 3B). These structural changes led us to examine the inhibitory parameters of GC376 with FIPV M^pro^ for comparison with SARS-CoV and SARS-CoV-2 M^pro^.

### GC376 Has Higher Affinity to Feline Infectious Peritonitis Virus M^pro^ Compared to SARS-CoV-2

**FIGURE 4:**
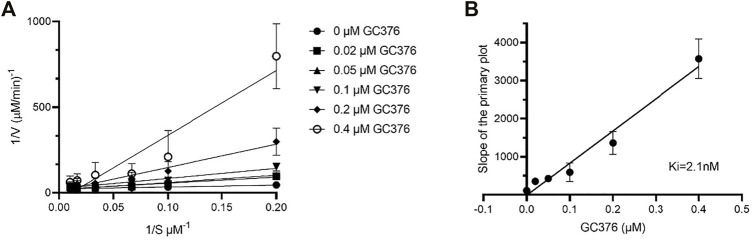
Determination of K_i_ values of GC376 with FIPV M^pro^. Lineweaver–Burk plot **(A)** and secondary plots of competitive inhibition **(B)**. Data are presented as mean ± SEM, *n* = 3.

IC_50_ and K_i_ values quantitatively reflect the potency and affinity of a drug and are therefore important parameters to consider when undergoing inhibitor design. First, we determined the catalytic parameters of FIPV M^pro^ using our synthetic peptide FRET-substrate (Supplementary Table S2) (Arutyunova et al., 2021). Interestingly, feline coronavirus protease exhibited 24 times slower catalytic turnover rate than M^pro^ of SARS-CoV-2 with the same substrate, and a lower K_m_ value. The K_i_ values for GC376 inhibition were determined to be 2.1 nM for FIPV M^pro^ (Figure 4), lower in comparison to previously determined K_i_ values of SARS-CoV and SARS-CoV-2 M^pro^, which were 20 and 40 nM, respectively (Table 1) (Vuong et al., 2020; Arutyunova et al., 2021).

**TABLE 1:** Comparison of K_i_ values of GC376 between FIPV M^pro^, SARS-CoV M^pro^, and SARS-CoV-2 M^pro^. Data are presented as mean ±SEM, *n* = 3.

Protease	Calculated Ki (nM)
FIPV M^pro^	2.1
SARS-CoV M^pro^ ^a^	20
SARS-CoV-2 M^pro^ ^a^	40

a Data from Arutyunova et al. (2021).

### Improved Derivatives of GC376 Are Also Potent Toward Feline Infectious Peritonitis Virus M^pro^

We recently demonstrated that derivatives of GC376 with singly or doubly modified constituents resulted in improved potency with SARS-CoV-2 M^pro^, having lower IC_50_ and EC_50_ values (Vuong et al., 2021). The singly modified compounds contain derivatives that include a cyclopropyl group (**1a**) in the P2 position where the S2 pocket typically recognizes a Leu residues side chain, and a 3-fluorobenzyl (**2c**) or 3-chlorophenylethyl group (**2d**) in the P3 position recognized by the S4 pocket (Table 2). The doubly modified compounds all included a cyclopropyl group in the P2 position, as well as a 3-fluorobenzyl (**2c**), 3-chlorophenylethyl **(2d)**, or 4-methoxyindole **(2e)** group at the P3 position (Table 3). In order to assess if these inhibitor derivatives also have improved potency with FIPV M^pro^ as they did with SARS-CoV-2 M^pro^, IC_50_ values were calculated and compared. *In vitro* analysis with purified FIPV M^pro^ revealed that the doubly modified inhibitor had stronger effects on IC_50_ values than a singly modified inhibitor, bringing the IC_50_ to the double-digit nanomolar range. This is a similar trend as seen with SARS-CoV-2 M^pro^ using the same doubly modified inhibitors. Overall, this suggests that inhibitors targeting FIPV M^pro^ can be improved and warrant further assessment in cellular and animal studies.

**TABLE 2:** Singly modified derivatives of GC373 at the P2 or P3 positions and their corresponding IC_50_ values. Data are presented as mean ± SEM, *n* = 3.

Entry	Structure	FIPV M^pro^ IC_50_ (µM)	SARS-CoV-2 M^pro^ IC_50_ (µM)^a^
**GC376**	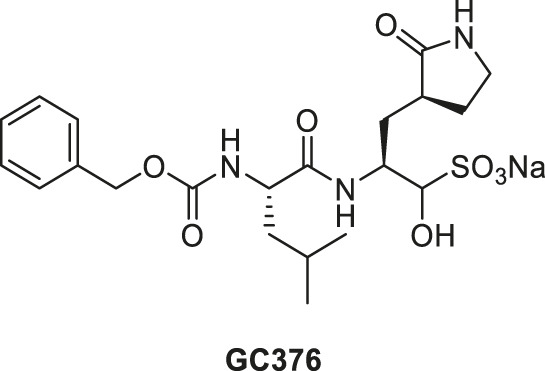	0.13 ± 0.02	0.19 ± 0.04
**1a**	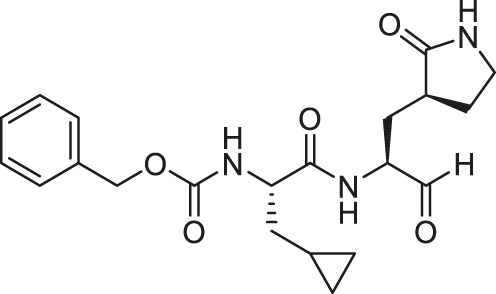	0.10 ± 0.07	0.05 ± 0.01
**1d**	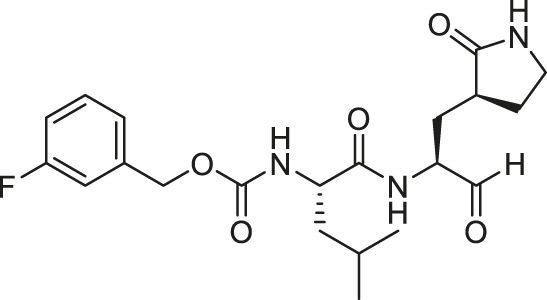	0.07 ± 0.01	0.13 ± 0.04
**1e**	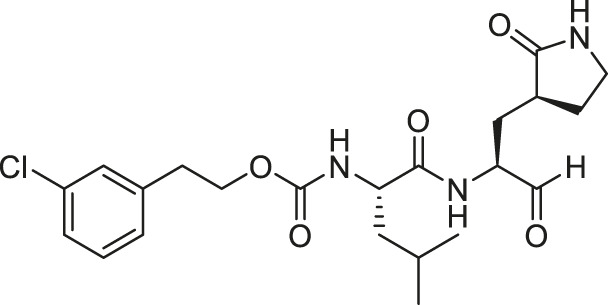	0.13 ± 0.02	0.15 ± 0.05
**1g**	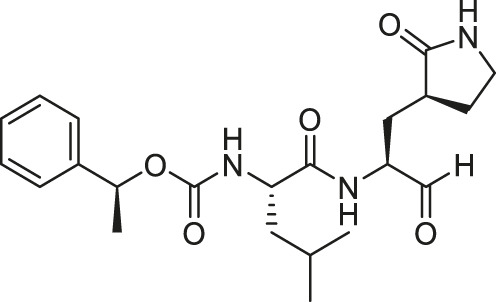	0.43 ± 0.09	0.27 ± 0.09

a Data from Vuong et al. (2021).

**TABLE 3:** Doubly modified derivatives of GC376 at the P2 and P3 positions and their corresponding IC_50_ values. Data are presented as mean ± SEM, *n* = 3.

Entry	Structure	FIPV M^pro^ IC_50_ (µM)	SARS-CoV-2 M^pro^ IC_50_ (µM)^a^
**GC376**	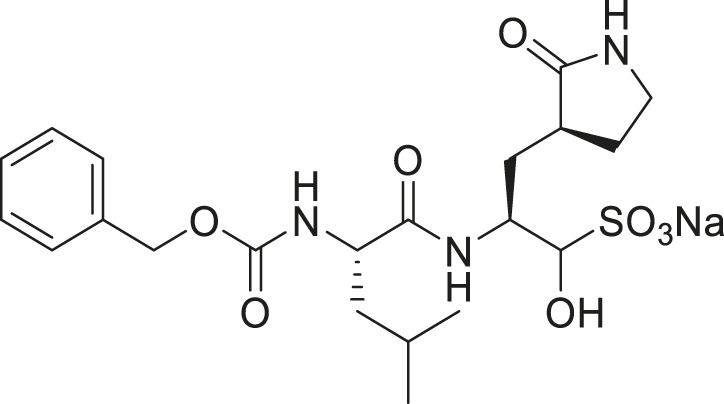	0.13 ± 0.02	0.19 ± 0.04
**2c**	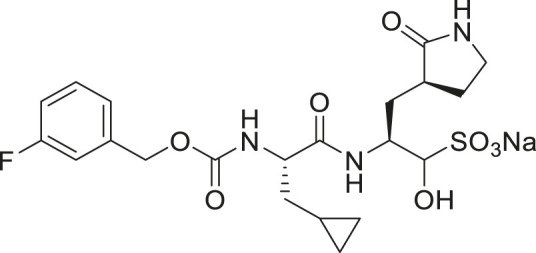	0.03 ± 0.01	0.07 ± 0.01
**2d**	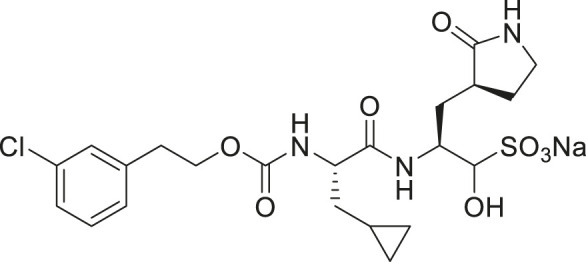	0.05 ± 0.02	0.08 ± 0.02
**2e**	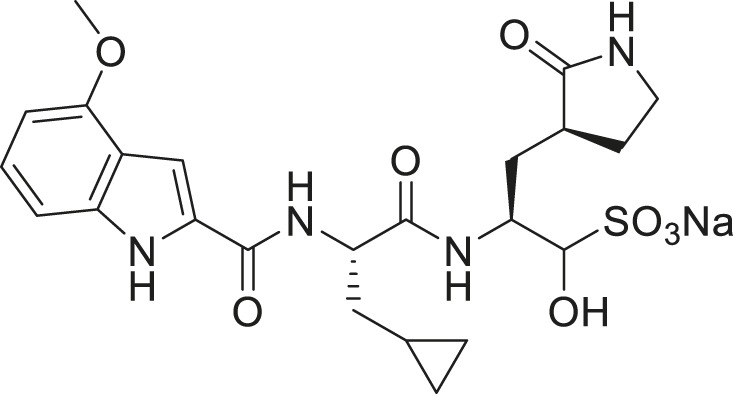	0.06 ± 0.02	0.04 ± 0.01

a Data from Vuong et al. (2021).

## Discussion

The FECV is commonly detected among domestic house cats and causes mild to no symptoms; however, mutations in FECV lead to FIP, a lethal systemic infection in cats. The M^pro^ inhibitor GC373 and its bisulfide aldehyde GC376 have been shown to treat the otherwise fatal infection in experimentally infected FIP cats, as well as naturally acquired FIP cats. Furthermore, GC376 has also been shown to be an effective inhibitor of other viral M^pro^ such as norovirus (PDB: 3UR9), transmissible gastric epidemic virus (PDB: 4F49), MERS-CoV (PDB: 5WTJ), porcine epidemic diarrhea virus (PDB: 6L70), SARS-CoV (PDB: 7LCQ), and more recently, SARS-CoV-2 (PDB: 6WTJ) (Tiew et al., 2011; Kim et al., 2015; Galasiti Kankanamalage et al., 2018; Vuong et al., 2020; Ye et al., 2020; Arutyunova et al., 2021). Our work here reports the crystal structure of GC376 and GC373 with FIPV M^pro^, allowing for its comparison with recent structures of viral proteases with these inhibitors and complementing the work done by others in developing the drug for treating FIP in cats.

The overall architecture of GC373 bound to FIPV M^pro^ is similar to other structures where the drug forms a C-shaped structure with pi stacking between the lactam ring and benzyl group in the P1 and P3 positions, respectively. While structures of SARS-CoV-2 M^pro^ co-crystallized with GC376 solved by other groups have shown the drug binding in both R and S hemithioacetal isomer conformations (Ma et al., 2020), here we only see the R conformation of the drug bound to FIPV M^pro^. We observe hydrogen binding of the oxyanion of GC373 to the general base His41 in FIPV M^pro^, similar to MERS-CoV and norovirus M^pro^ (Kim et al., 2012; Galasiti Kankanamalage et al., 2018). Nonetheless, this binding is in contrast to SARS-CoV, SARS-CoV-2, and PEDV M^pro^, where the oxyanion is bound by traditional backbone residues of the oxyanion hole (Kim et al., 2012; Lee et al., 2020; Arutyunova et al., 2021). Overall, this suggests flexibility in the binding between the active site residues and inhibitor, and further highlights the feline drugs' broad specificity.

We have previously shown that the N-terminal tail of M^pro^ plays a role in dimerization and drug stabilization (Arutyunova et al., 2021). The FIPV M^pro^-GC373 complex reveals weak hydrogen bonding of the hydroxyl group of Ser1 (protomer B) with the cyclic glutamine analog in GC373, bound to the active site of protomer A, providing additional coordination for the inhibitor. By contrast, no interaction is observed between Ser1 and GC373 in SARS-CoV-2 M^pro^. This led us to compare the inhibitory parameters of GC376 between the two M^pro^ to determine if these structural differences lead to improved drug binding. We recently showed that GC376 was an effective inhibitor of SARS-CoV M^pro^ and SARS-CoV-2 M^pro^ with K_i_ values of 20 and 40 nM, respectively (Table 1). In comparison, GC376 inhibited the FIPV M^pro^ with a K_i_ of 2.1 nM, 20 times higher in affinity than SARS-CoV-2 M^pro^. Together, the difference in K_i_ values further reflects structural plasticity among various M^pro^ that results in differences in how the drug binds the active site and thus affecting drug potency.

In order to increase the potency of GC376, our team has recently developed modified derivatives which showed lower IC_50_ values for SARS-CoV-2 M^pro^ compared to the parent compound (Vuong et al., 2021). The modification of P2 was chosen to be lipophilic since our previous crystal structures demonstrate that the S2 pocket responsible for binding the leucine moiety was mostly hydrophobic. The modification of the P3 position allowed for enhanced dipole interactions with the S4 pocket of the enzyme, potentially contributing to the higher affinity (Wang et al., 2016). Importantly, these derivatives, in particular the ones with double modifications, exhibit lower IC_50_ values with FIPV M^pro^ compared to SARS-CoV-2 M^pro^. Moving forward, enhanced drugs are needed for both FIPV infections as well as other coronavirus-related outbreaks. This crystal structure of FIPV M^pro^ in complex with GC376 will assist us in accelerating development of new derivatives to be used in clinical trials as broad-spectrum antivirals.

## Data Availability

The original contributions presented in the study are included in the article/Supplementary Material, and further inquiries can be directed to the corresponding author.
