# Supplementary material for: Crystallization of Feline Coronavirus Mpro With GC376 Reveals Mechanism of Inhibition
Source: Front Chem. 2022 Feb 24;10:852210. doi: 10.3389/fchem.2022.852210 (PMC8907848; doi:10.3389/fchem.2022.852210)
Supplement: Supplementary file 1 [file DataSheet1.docx]

**Supplementary Material**

**(A)**


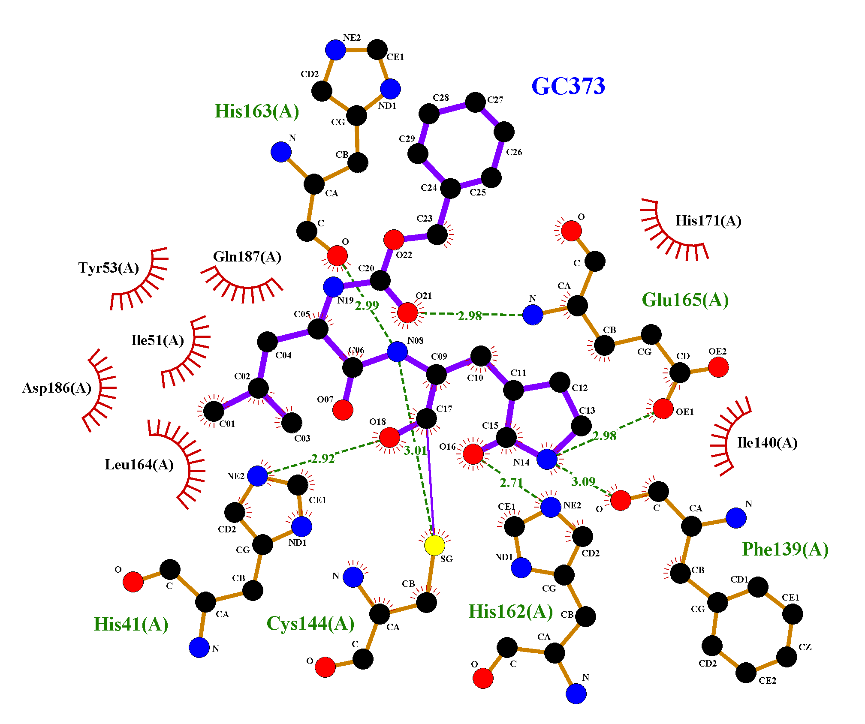


**(B)**


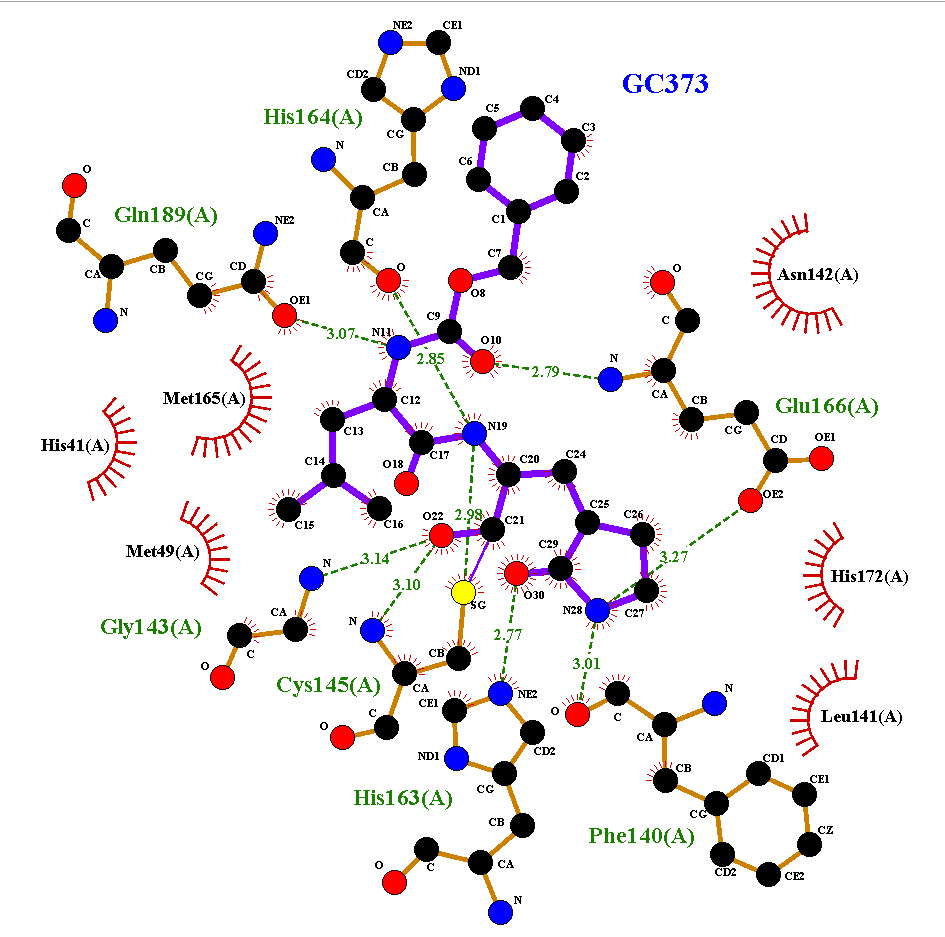


**Supplemental Figure 1.** LigPlot showing the hydrogen bond network between A) FIPV M^pro^ coordinating with GC373 (PDB: 7SNA) and B) SARS-CoV-2 M^pro^ coordinating with GC373 (PDB: 6WTK) using LigPlot^+^.

**(A)**


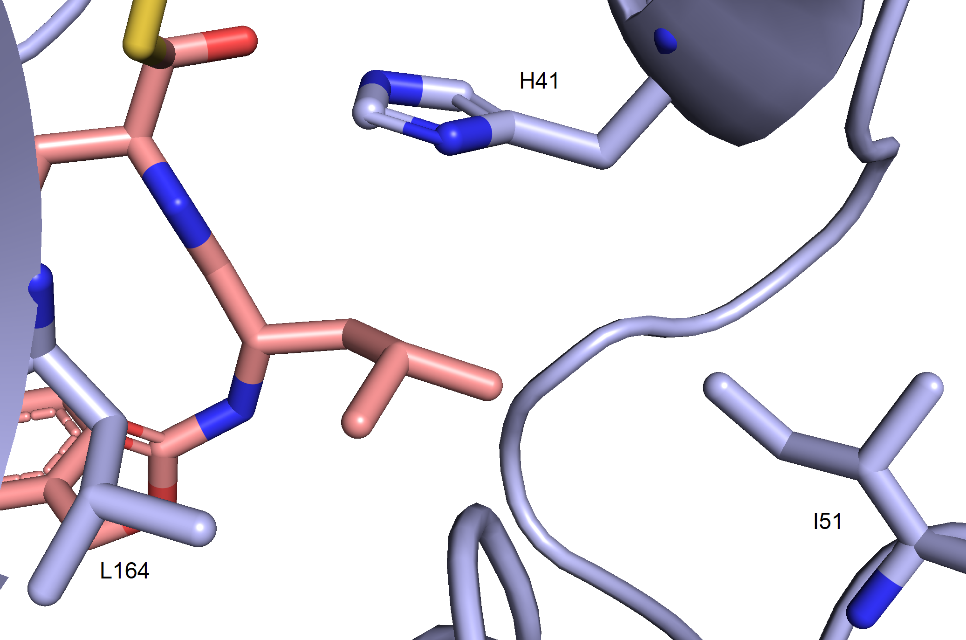


P2

**(B)**


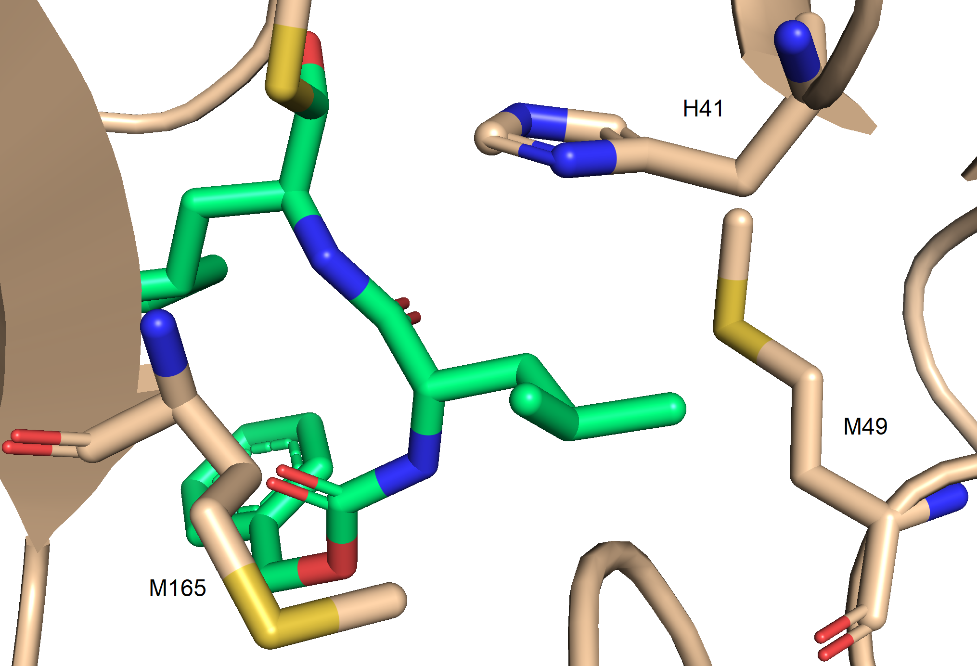


P2

**Supplemental Figure 2.** The Leu moiety at the P2 position of GC373/GC376 is supported by hydrophobic interactions within the S2 pocket. A) The S2 pocket of FIPV M^pro^ (PDB: 7SNA) is formed by residues His41, Ile51 and Leu164. The S2 pocket of SARS-CoV-2 M^pro^ (PDB: 6WTK) is formed by residues His41, Met49 and Met165.


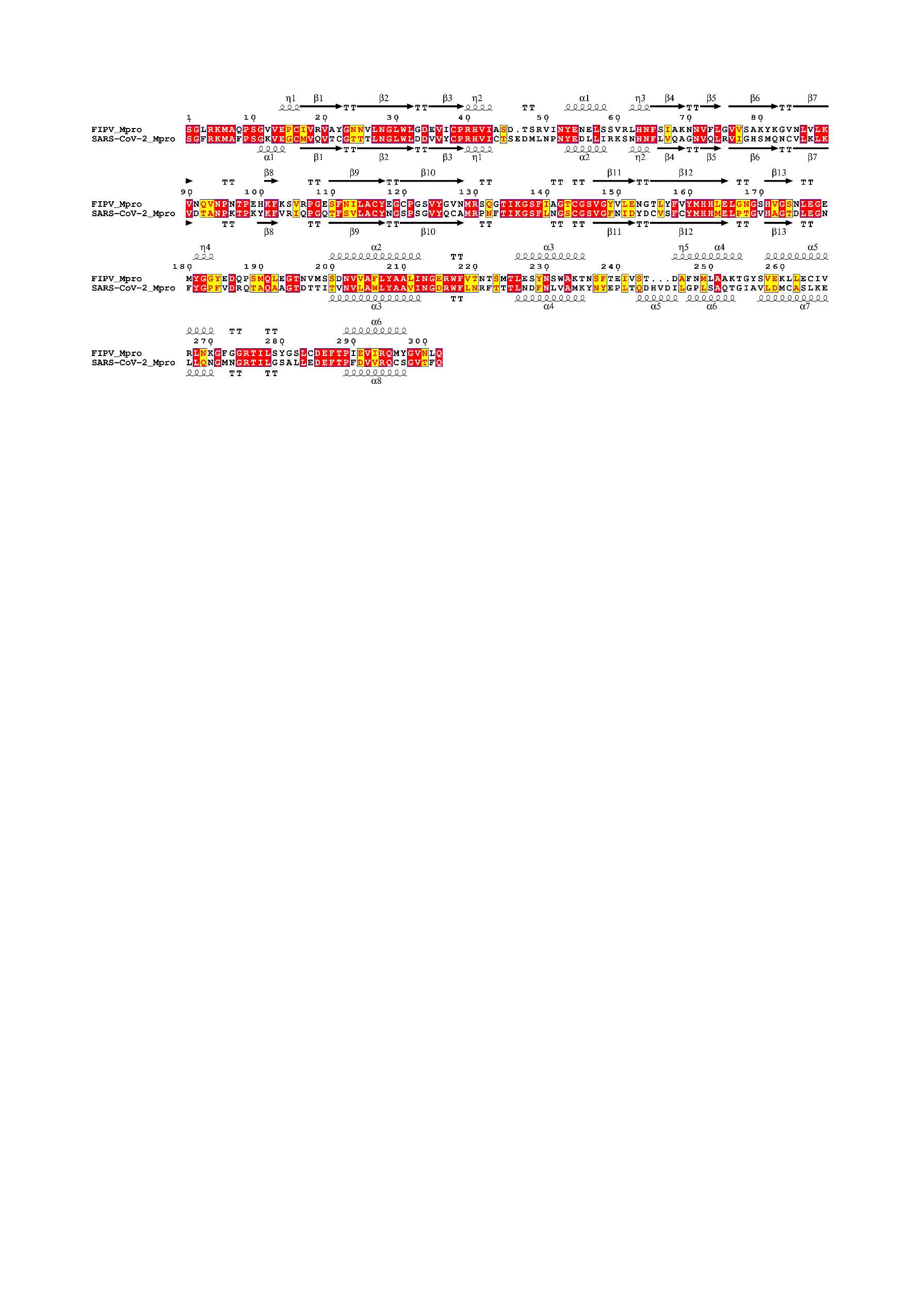


**Supplemental Figure 3.** Secondary sequence alignment of: FIPV M^pro^ bound to GC376 (PDB: 7SMV) compared to SARS-CoV-2 bound to GC376 (PDB: 6WTJ) using ESPript 3.0. Sequence identity is 44% while sequence conservation is 60%.

**Supplemental Table 1.** Summary of X-ray data collection and refinement statistics, molecular replacement. Values in parentheses are for the highest-resolution shell.

|  | FIPV_GC373 | FIPV_GC376 |  |
| --- | --- | --- | --- |
| PDB entry  **Data collection** | 7SNA | 7SMV |  |
| Space group | C2 | P2_1_ 2_1_ 2_1_ |  |
| Cell dimensions |  |  |  |
| *a*, *b*, *c* (Å) | 110.66 66.74 79.07 | 67.8 77.42 111.75 |  |
| α, β, γ (°) | 90 91.06 90 | 90 90 90 |  |
| Resolution (Å) | 44.94 - 2.05 (2.123 - 2.05) | 45.31 - 1.93 (1.999 - 1.93) | |
| Observations | 243762 (24081) | 591081 (59672) |  |
| *R*_merge_ | 0.08867 (1.097) | 0.1019 (3.484) |  |
| *I* / σ*I* | 15.94 (1.86) | 18.52 (0.85) |  |
| Completeness (%) | 99.74 (99.39) | 99.90 (99.66) |  |
| Redundancy | 6.6 (6.7) | 13.2 (13.4) |  |
| CC1/2 | 99.90 (75.50) | 99.90 (38.80) |  |
|  |  |  |  |
| **Refinement** |  |  |  |
| Resolution (Å) | 44.94 - 2.05 | 45.31 - 1.93 |  |
| No. reflections | 36225 | 44906 |  |
| *R*_work_ / *R*_free_ | 17.45/22.73 | 19.12/24.44 |  |
| No. atoms | 5117 | 4940 |  |
| Protein | 4623 | 4598 |  |
| Ligand/ion | 120 | 118 |  |
| Water | 436 | 284 |  |
| *B*-factors | 35.56 | 46.42 |  |
| Protein | 34.96 | 46.45 |  |
| Ligand/ion | 30.95 | 41.52 |  |
| Water | 42.59 | 46.92 |  |
| R.m.s. deviations |  |  |  |
| Bond lengths (Å) | 0.013 | 0.018 |  |
| Bond angles (°) | 1.19 | 2.16 |  |

*Values in parentheses are for highest-resolution shell. Each data set was collected from single crystal

**Supplemental Table** **2**. Comparison of catalytic parameters of FIPV M^pro^, SARS-CoV M^pro^, and SARS-CoV-2 M^pro^ using the same peptide-based FRET substrate. Data is presented as mean ± SEM, *n*=3.

| **Protease** | **Calculated K_i_ (nM)** | **K_cat_ (min^-1^)** | **K_cat_/K_0.5_ (min­^-1^uM^-1^)** |
| --- | --- | --- | --- |
| FIPV M^pro^ | 5.6 ± 1.8 | 5.7 ± 1.4 | 1.0 ± 0.1 |
| SARS-CoV M^pro*^ | 70 ± 10 | 135 ± 6 | 1.8 ± 0.4 |
| SARS-CoV-2 M^pro*^ | 52 ± 17 | 30 ± 2 | 0.6 ± 0.2 |

^*^Data from Arutyunova et al., 2021.

**(A)**


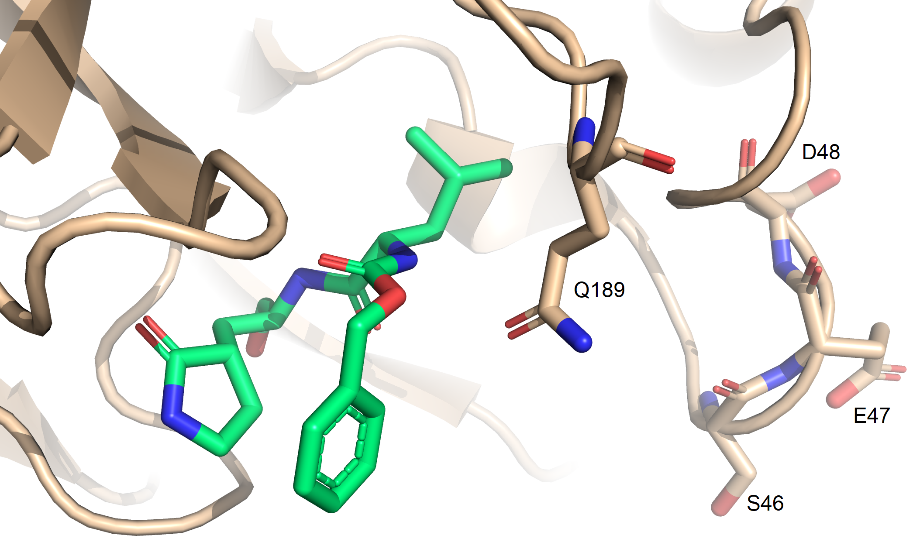


P2

**(B)**


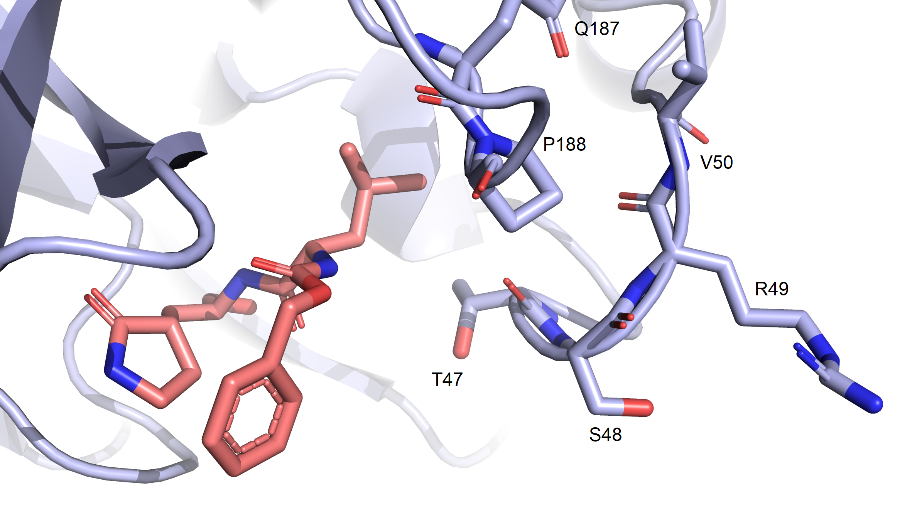


P2

**Supplemental Figure 4.** Stabilization of the backbone of GC373. A) Gln189 of SARS-CoV-2 M^pro^ stabilizes the GC373 dipeptide backbone (PDB: 6WTK). B) An unstructured loop in FIPV M^pro^ fits into the space between the S3 and S4 pocket to form hydrophobic interactions, further supporting binding of the GC373 (PDB:7SNA).

**
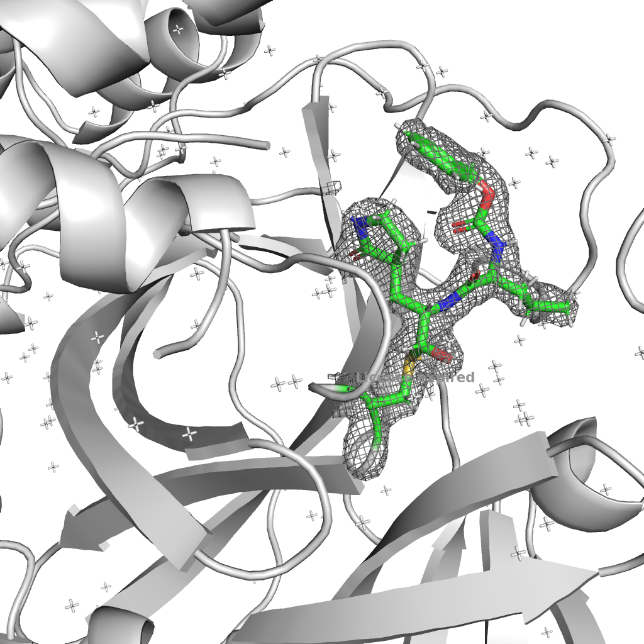

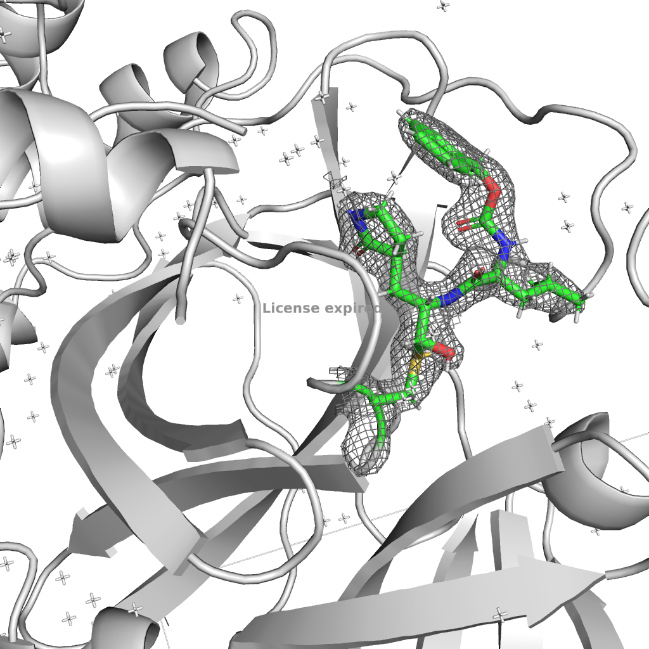
**

**Supplemental Figure 5.** GC373 (left) and GC376 (right) covalently binds the active site cysteine 144 of FIPV M^pro^. Electron density shown in gray mesh at 1σ.

**
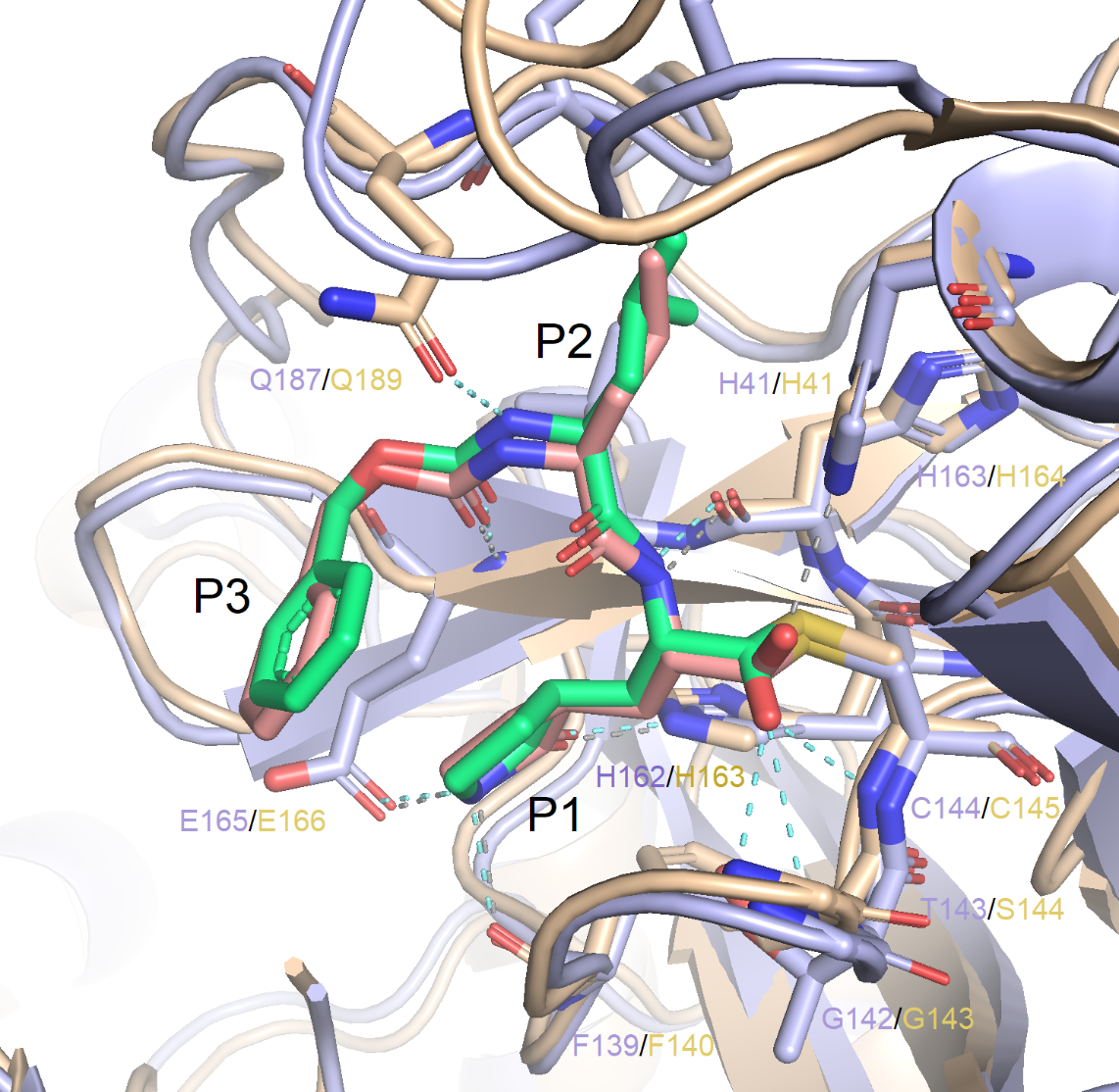
**

**Supplemental Figure 6.** Overlay of FIPV M^pro^ bound to GC373 in lavender (PDB: 7SNA) and SARS-CoV M^pro^ bound to GC373 in tan (PDB: 6WTK) reveals differences in the drug binding in the active site between the two M^pro^. Dashed grey lines represent hydrogen bonding between GC373 and FIPV M^pro^ and dashed blue lines represent hydrogen bonding between GC373 and SARS-CoV-2 M^pro^.
